# Involvement of conserved tryptophan residues for secretion of TIMP-2

**DOI:** 10.3892/ol.2013.1771

**Published:** 2013-12-23

**Authors:** TAMAMI UKAJI, YUKIKO SASAZAWA, KAZUO UMEZAWA, SIRO SIMIZU

**Affiliations:** 1 Department of Applied Chemistry, Faculty of Science and Technology, Keio University, Yokohama, Kanagawa 223-8522, Japan; 2 Department of Molecular Target Medicine Screening, Aichi Medical University School of Medicine, Nagakute, Aichi 480-1195, Japan

**Keywords:** tissue inhibitor of metalloproteinase, secretion, conserved tryptophan

## Abstract

Tissue inhibitor of metalloproteinases (TIMPs) are endogenous inhibitor proteins of matrix metalloproteinases and contain 12 cysteine residues that are conserved among TIMPs, and which are important for their activity and structure. In the present study, three tryptophan residues conserved among TIMPs were revealed to be important for the secretion of TIMP-2. Replacement of conserved tryptophan residues in TIMP-2 with alanine led to a decrease in extracellular TIMP-2 levels and an increase in intracellular TIMP-2 levels. Furthermore, wild-type TIMP-2 and TIMP-2 mutated at unconserved tryptophan residues mainly localized in the Golgi apparatus, while TIMP-2 proteins mutated at conserved tryptophan were mainly observed in the endoplasmic reticulum (ER). This indicated that conserved tryptophan is essential for transporting TIMP-2 from the ER to Golgi apparatus. These observations suggested that conserved tryptophan residues among the TIMP family of proteins have critical roles for ER-Golgi transport and subsequent secretion of TIMP-2.

## Introduction

The extracellular matrix (ECM) is a complex network composed of macromolecules, including collagen, hyaluronic acid, fibronectin, proteoglycans and glycoproteins, which presents within all tissues and organs and is necessary for multicellular organisms to maintain cellular function. A number of ECM components are degraded by matrix metalloproteinases (MMPs), a family of zinc-dependent endopeptidases. MMPs are involved in a number of diseases, including tumor metastasis, rheumatoid arthritis and periodontal disease (1). Tissue inhibitors of metalloproteinases (TIMPs) are endogenous inhibitors of MMPs and contribute to inhibiting tumorigenesis and subsequent malignant progression by regulating ECM turnover (2–4). TIMPs bind to the active sites of MMPs non-covalently in a 1:1 stoichiometric manner and constitute a family of four proteins (TIMP-1, -2, -3 and -4). Mammalian TIMP family proteins are subdivided into N- and C-terminal subdomains consisting of ~125 and ~65 amino acids, respectively (5). Each domain contains three disulfide bonds formed between the cysteine residues conserved among all four TIMPs, which are important for their activity and structure (6). The N-terminal domain is highly conserved among four human TIMPs and in TIMPs of other species, and acts as a depressant of MMPs and specific a disintegrin and metalloproteinase (ADAM) and a disintegrin and metalloproteinase with thrombospondin motif (ADAMTS) family members, while the C-terminal domain mediates protein-protein interaction (7).

Although the four TIMPs exhibit similar structures, they are expressed in various tissues and have various MMP-inhibitory profiles (5). Among them, TIMP-2 has the unique feature of biphasic regulation of MMPs. TIMP-2 inhibits all active MMPs, by contrast, it also regulates the MT1-MMP-dependent activation of pro-MMP2 (8,9). Thus, TIMP-2 has two different aspects of MMP-regulating activity. TIMP-2 also inhibits endothelial cell proliferation and angiogenesis independently from MMP regulating activity (10).

The present study focused on the highly conserved tryptophan residues in TIMP-2 and investigated whether they are important for their function.

## Materials and methods

### Cell culture

A human fibrosarcoma HT1080 cell line obtained from Japanese Cancer Research Resources Bank (Tsukuba, Japan) was cultured in DMEM supplemented with 10% (v/v) fetal bovine serum, 100 U/ml penicillin G, 100 mg/l kanamycin, 600 mg/l L-glutamine and 2.25 g/l NaHCO_3_ at 37°C in a humidified incubator with 5% CO_2_.

### Construction of TIMP-2 expression plasmid and site-directed mutagenesis

The human TIMP-2 gene was amplified from the cDNA of HT1080 cells and subcloned into a pCI-neo vector (Promega Corporation, Madison, WI, USA). Certain tryptophan residues in TIMP-2 were substituted with alanine residues by PCR site-directed mutagenesis using overlap extension technique. The sequences of primers used for the mutagenesis were as follows: W133A forward, 5′-CTTCATCGT GCCCGCGGACACCCTGAGCACC-3′ and reverse, 5′-GGT GCTCAGGGTGTCCGCGGGCACGATGAAG-3′; W174A forward, 5′-GACGAGTGCCTCGCGATGGACTGGGTC-3′ and reverse, 5′-GACCCAGTCCATCGCGAGGCACTC GTC-3′; W177A forward, 5′-GCCTCTGGATGGACGCGG TCACAGAGAAG-3′ and reverse, 5′-CTTCTCTGTGACCGC GTCCATCCAGAGGC-3′; and W203A forward, 5′-CGGCTC CTGTGCGGCGTACCGCGGCGCGGCGC-3′ and reverse, 5′-GCGCCGCGCCGCGGTACGCCGCACAGGAGCCG-3′.

### Establishment of TIMP-2-overexpressing stable cell lines

The permanent cell lines stably expressing wild-type (wt) and mutant TIMP-2-myc-his6 (T2-MH) were established by transfecting the vectors into HT1080 cells using Lipofectamine LTX (Life Technologies, Carlsbad, CA, USA) followed by G418 (Roche Diagnostics, Indianapolis, IN, USA) selection. The clone cells that expressed high levels of myc-his6-tagged wt TIMP-2 and TIMP-2 W133A, W174A, W177A and W203A were designated as HT1080-T2-MH, HT1080-T2-MH/W133A, HT1080-T2-MH/W174A, HT1080-T2-MH/W177A and HT1080-T2-MH/W203A cells, respectively. The cells transfected with pCI-neo were designated as HT1080-neo.

### RNA isolation and semi-quantitative polymerase chain reaction (PCR) analysis

Total RNAs were extracted from cultured cells by using TRIzol reagent (Life Technologies). Reverse transcription was performed at 37°C for 120 min with a High Capacity cDNA Reverse Transcription kit (Life Technologies). The cDNA was used for PCR amplification with rTaq DNA polymerase (Takara Bio, Inc., Shiga, Japan). The number of PCR cycles for each product was determined following confirmation of the efficacy of amplification and having defined the linear primers used for semi-quantitative PCR. The number of cycles and annealing temperatures were as follows: Exogenous T2-MH forward, 5′-GGCGTTTTG CAATGCAGATGTAGTG-3′ and reverse, 5′-GTGATGGTGAT GATGCAGATCCTCTTCTGAGATGAG-3′ (25 cycles; 55°C); and GAPDH forward, 5′-TGAAGGTCGGAGTCAACG GATTTGGT-3′ and reverse, 5′-CATGTGGGCCATGAGGTC CACCAC-3′ (25 cycles; 55°C). PCR products were electrophoresed on 1% agarose gels, stained with ethidium bromide and visualized with a UV illuminator (Desktop Gel Imager SCOPE21; OPTIMA Inc., Tokyo, Japan).

### Western blot analysis

Western blot analysis was performed as previously described with slight modifications (11). For detection of intracellular protein levels, cells were lysed with lysis buffer [50 mM Tris-HCl (pH 7.5), 150 mM NaCl, 0.1% (w/v) SDS, 1% (v/v) Triton X-100, 1% (w/v) sodium deoxycholate and 1 mM PMSF] and centrifuged at 14,000 × g for 10 min. The protein concentrations of the supernatants were determined and secreted proteins were collected from conditioned media. Aliquots of the cell lysates with 6X sample buffer [350 mM Tris-HCl (pH 6.8), 30% glycerol, 0.012% bromophenol blue, 6% SDS and 30% 2-Mercaptoethanol] were subsequently boiled for 3 min and electrophoresed on SDS-polyacrylamide gels. Proteins were transferred to polyvinylidene difluoride membranes and immunoblotted with anti-c-myc (Santa Cruz Biotechnology, Inc., Santa Cruz, CA, USA) or anti-β-actin (Sigma-Aldrich, St Louis, MO, USA) antibodies. Detection was performed with enhanced chemiluminescence reagent (EMD Millipore Corporation, Billerica, MA, USA).

### Immunofluorescence analysis

Immunofluorescence analysis was performed as previously described with slight modifications (12). The cells grown on cover slips were washed with phosphate-buffered saline (PBS), fixed with 4% para-formaldehyde and permeabilized with 0.1% Triton X-100 for 10 min. Following blocking with 2% bovine serum albumin, the cells were incubated with anti-c-myc (Santa Cruz Biotechnology, Inc. or Cell Signaling Technology, Inc., Beverly, MA, USA), anti-KDEL (StressGen Bioreagents, Victoria, BC, Canada) and anti-GRASP65 (Santa Cruz Biotechnology, Inc.) antibodies for 1 h. Alexa 488-conjugated anti-mouse and anti-rabbit IgG (Life Technologies) were used as the secondary antibodies. After washing three times with PBS, the cells were incubated with 2 μg/ml Hoechst 33258 (Wako Pure Chemical Industries, Ltd., Osaka, Japan) for 10 min to stain the nuclei. Then, the cells were washed three times with PBS and observed under a fluorescence microscope (EVOS FL Cell Imaging System; Life Technologies).

## Results

### Conserved tryptophan residues in TIMP-2 are important for its secretion

**Figure 1:**
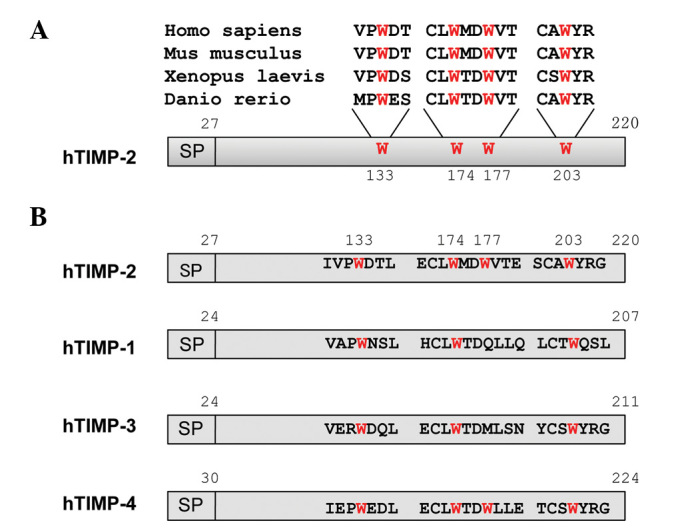
Schematic illustration of amino acid sequence homology among TIMP family proteins. (A) Conserved TIMP-2 sequences in *Homo sapiens*, *Mus musculus*, *Xenopus laevis* and *Danio rerio*, with tryptophan residues indicated in red. (B) Conserved sequences of TIMP family proteins, with tryptophan residues indicated in red. TIMP, tissue inhibitor of metalloproteinase; SP, single peptide.

**Figure 2:**
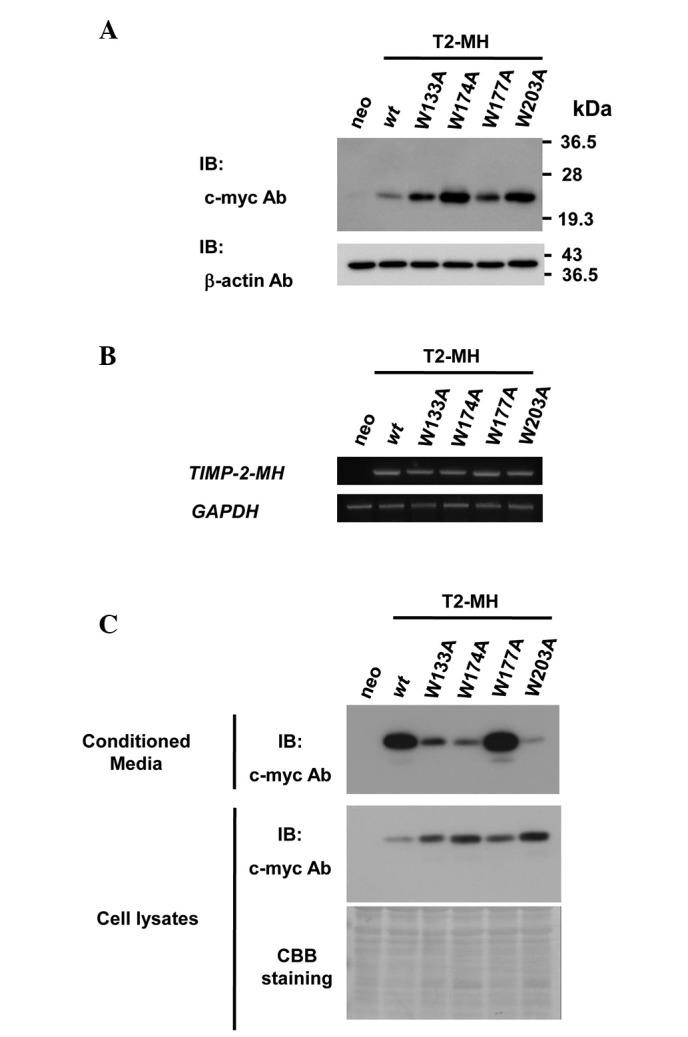
Conserved tryptophan residues in TIMP-2 are important for its secretion. (A) HT1080-neo, HT1080-T2-MH, HT1080-T2-MH/W133A, HT1080-T2-MH/W174A, HT1080-T2-MH/W177A and HT1080-T2-MH/W203A cells were lysed and aliquots of the cell lysates were electrophoresed and immunoblotted with anti-c-myc or anti-β-actin antibodies. (B) Total RNAs were isolated from each cell line and exogenous TIMP-2 mRNA levels were analyzed by semi-quantitative polymerase chain reaction. (C) Each cell line was cultured in serum-free media for 24 h. Samples from the conditioned media and aliquots of the cell lysates were electrophoresed and immunoblotted with anti-c-myc antibody. CBB staining was used as a loading control. TIMP-2, tissue inhibitor of metalloproteinase-2; T2-MH, TIMP-2-myc-his6; IB, immunoblotting; CBB, Coomassie brilliant blue.

There are four tryptophan residues (Trp^133^, Trp^174^, Trp^177^ and Trp^203^) in human TIMP-2 and they are highly conserved in vertebrates, as shown in Fig. 1A. However, although three of these tryptophan residues (Trp^133^, Trp^174^ and Trp^203^) are conserved among human TIMP family proteins, one (Trp^177^) is unconserved, as shown in Fig. 1B. In order to demonstrate the importance of conserved tryptophan residues for TIMP-2 secretion, cell lines overexpressing C-terminus myc-his6 tagged wt TIMP-2 (T2-MH) or mutants replacing tryptophan residues to alanine residues (T2-MH/W133A, T2-MH/W174A, T2-MH/W177A and T2-MH/W203A) were established (Fig. 2A). Equal amounts of exogenous TIMP-2 mRNA in the stable cell lines were confirmed by semi-quantitative PCR analysis (Fig. 2B).

Since TIMPs are secreted and function in the extracellular space, the effect of the mutation of tryptophan residues on the secretion of TIMP-2 was first examined. The levels of secreted wt and W177A mutant in conditioned media were approximately the same, whereas the protein levels of secreted W133A, W174A and W203A mutants were significantly lower than those of the wt (Fig. 2C). These results indicated that conserved tryptophan residues are required for TIMP-2 secretion.

### Conserved tryptophan residues in TIMP-2 are important for its endoplasmic reticulum (ER)-Golgi traffic

**Figure 3:**
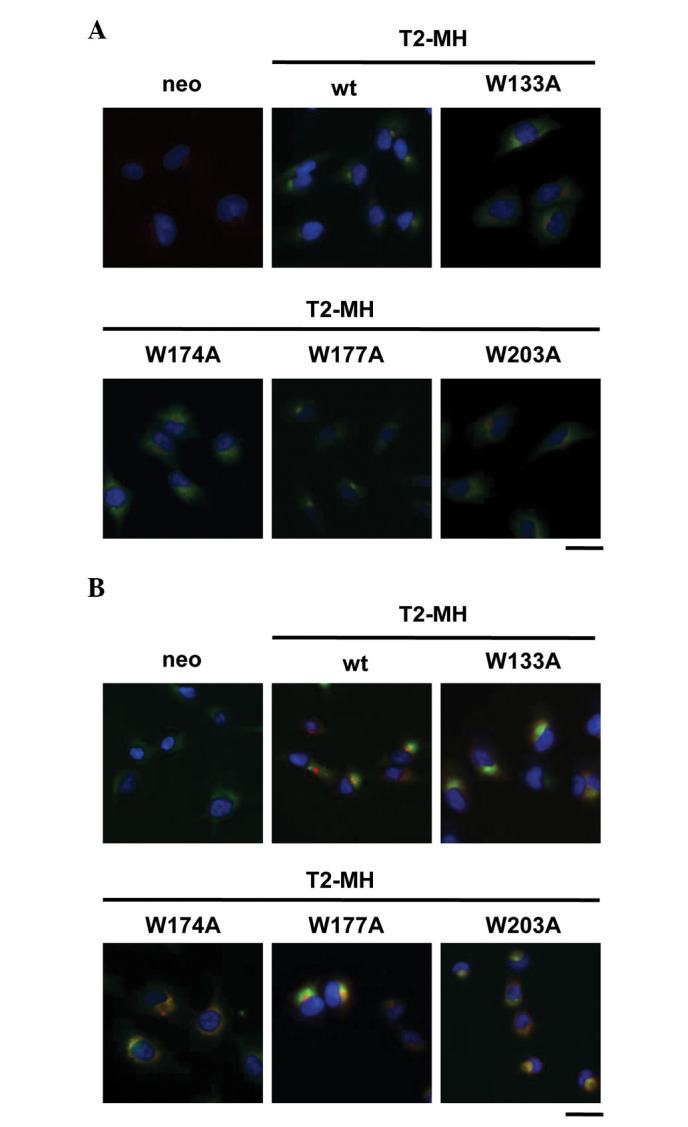
Role of conserved tryptophan residues of TIMP-2 on the intracellular localization. (A) HT1080-neo, HT1080-T2-MH, HT1080-T2-MH/W133A, HT1080-T2-MH/W174A, HT1080-T2-MH/W177A and HT1080-T2-MH/W203A cells were fixed and stained with Hoechst 33258 (nuclei; blue) and anti-c-myc (T2-MH; green) and anti-GRASP65 (Golgi apparatus; red) antibodies. (B) HT1080-neo, HT1080-T2-MH, HT1080-T2-MH/W133A, HT1080-T2-MH/W174A, HT1080-T2-MH/W177A and HT1080-T2-MH/W203A cells were fixed and stained with Hoechst 33258 (nuclei; blue) and anti-c-myc (T2-MH; red) and anti-KDEL (endoplasmic reticulum; green) antibodies. Cells were observed by fluorescence microscopy (scale bars, 25 μm). TIMP-2, tissue inhibitor of metalloproteinase-2; T2-MH, TIMP-2-myc-his6; wt, wild type.

The majority of secretory proteins contain hydrophobic signal peptides that direct proteins to the ER and Golgi apparatus and, subsequently, to the extracellular space or plasma membrane through the ER-Golgi secretory pathway (13). To investigate whether the lower levels of secreted TIMP-2 mutated in conserved tryptophan residues was due to the inhibition of ER-Golgi transport, the effect of the mutation of conserved tryptophan residues on the intracellular localization of TIMP-2 was examined. Immunofluorescence analysis revealed that the wt and W177A mutant stained with anti-c-myc antibody were mainly localized in the Golgi apparatus (Fig. 3A), whereas the W133A, W174A and W203A mutants were mainly localized in the ER (Fig. 3B). These results indicated that the mutation in conserved tryptophan residues inhibited the ER-Golgi traffic of TIMP-2. Thus, conserved tryptophan residues of TIMP-2 are critical for transportation to the Golgi apparatus and subsequent secretion.

## Discussion

Previous studies have shown that TIMPs contain 12 conserved cysteine residues that are important for its structure (6). Since the conservation of the relative positions of these residues may support their function and structure, the present study focused on the conserved tryptophan residues of TIMP-2. The observations revealed that the conserved tryptophan residues of TIMP-2 are important in its ER-Golgi transport and subsequent secretion. Furthermore, the importance of conserved tryptophan residues in other family proteins (TIMP-1, -3 and -4) for their secretion was evaluated (data not shown). Besides three conserved tryptophan residues, TIMP-1 has no unconserved tryptophan residue, whereas TIMP-3 and -4 contain one unconserved tryptophan residue. Using HT1080 stable cell lines overexpressing mutant proteins, in which each tryptophan residue is replaced by alanine, the conserved tryptophan residues in TIMP-1, -3 and -4 were demonstrated to be essential for secretion, as in the case of TIMP-2. Thus, it was demonstrated that tryptophan residues conserved among TIMP family proteins are critical for the secretion of TIMP family proteins. However, the mechanisms underlying the inhibitory effect on ER-Golgi transport due to the mutation of conserved tryptophan residues remain unclear. One possible reason is the inhibition of the glycosylation of TIMP-2 by the mutation of tryptophan residues, since TIMP-2 contains a Trp-Xaa-Xaa-Trp (where Xaa represents any amino acid) sequence that is the consensus sequence for C-mannosylation (14) and Trp^174^ is a potential C-mannosylation site. In the current study, to examine whether TIMP-2 is C-mannosylated at Trp^174^, recombinant TIMP-2 protein was purified from conditioned medium of HT1080-TIMP-2-MH cells and purified TIMP-2 was analyzed by MALDI-TOF MS. However, no peak of fragments generated by C-mannosylation was observed, suggesting that C-mannosylation is not involved in the secretion of TIMP-2.

The majority of secretory proteins are transported from the ER to the Golgi apparatus by the membrane vesicles composed of a multisubunit protein complex, coat protein complex II (COP-II). In this process, cargo proteins are sorted by the Sec24 subunit of COP-II and are incorporated into COP-II vesicles for transportation from the ER to the Golgi apparatus (13). It is likely that the mutation of tryptophan to alanine changes the conformation of TIMPs and causes abnormalities of recognition by the proteins involved in the step of ER-Golgi transport.

Increasing evidence indicates that the TIMP family of proteins are involved in the suppression of tumor invasion and metastasis in numerous types of human cancer by regulating ECM turnover (2–4). Therefore, the observations of the current study provide a new insight into the TIMP family of proteins and are likely to contribute to understanding the mechanisms of cancer metastasis.
